# The Interpretation of Cholesterol Balance Derived Synthesis Data and Surrogate Noncholesterol Plasma Markers for Cholesterol Synthesis under Lipid Lowering Therapies

**DOI:** 10.1155/2017/5046294

**Published:** 2017-02-22

**Authors:** Frans Stellaard, Dieter Lütjohann

**Affiliations:** Institute for Clinical Chemistry and Clinical Pharmacology, University Clinics of Bonn, Bonn, Germany

## Abstract

The cholesterol balance procedure allows the calculation of cholesterol synthesis based on the assumption that loss of endogenous cholesterol via fecal excretion and bile acid synthesis is compensated by* de novo* synthesis. Under ezetimibe therapy hepatic cholesterol is diminished which can be compensated by hepatic* de novo* synthesis and hepatic extraction of plasma cholesterol. The plasma lathosterol concentration corrected for total cholesterol concentration (R_Lath) as a marker of* de novo* cholesterol synthesis is increased during ezetimibe treatment but unchanged under treatment with ezetimibe and simvastatin. Cholesterol balance derived synthesis data increase during both therapies. We hypothesize the following. (1) The cholesterol balance data must be applied to the hepatobiliary cholesterol pool. (2) The calculated cholesterol synthesis value is the sum of hepatic* de novo* synthesis and the net plasma—liver cholesterol exchange rate. (3) The reduced rate of biliary cholesterol absorption is the major trigger for the regulation of hepatic cholesterol metabolism under ezetimibe treatment. Supportive experimental and literature data are presented that describe changes of cholesterol fluxes under ezetimibe, statin, and combined treatments in omnivores and vegans, link plasma R_Lath to liver function, and define hepatic* de novo* synthesis as target for regulation of synthesis. An ezetimibe dependent direct hepatic drug effect cannot be excluded.

## 1. Introduction

Plasma cholesterol (chol) and in particular LDL-chol can be lowered with drug treatments reducing chol absorption (ezetimibe, EZE), chol synthesis (statins), or both. Under normal treatment conditions EZE reaches the smallest plasma chol reduction followed by statin treatment and the combination treatment with the largest plasma chol reduction [1]. The lower efficiency of EZE can be explained by the induction of increased chol synthesis as a reaction on the reduced chol absorption rate [2]. Recently we published a paper on the validity of surrogate markers for chol absorption, chol synthesis and bile acid synthesis [3] in which the data from two previously published studies were reevaluated [1, 4]. Reference [1] dealt with a randomized, double-blind, placebo-controlled, four-period, balanced crossover study in omnivore subjects treated with placebo (PLAC), EZE, simvastatin (SIMVA), and a combination (EZE + SIMVA) during 7 weeks of treatment. Chol absorption, synthesis, and catabolism were measured. Reference [4] dealt with a similar study design in which the same parameters were measured in healthy vegans under PLAC and EZE treatment. The results led to some interesting observations that need more discussion in order to understand the mechanisms by which EZE and statins induce a reduction in plasma chol concentrations and evaluate the interpretation of cholesterol balance derived chol synthesis values. The following observations were made concerning chol synthesis as was measured by the cholesterol balance technique and by the plasma lathosterol/cholesterol concentration ratio (R_Lath).

1. EZE led to a strongly increased chol synthesis, a mildly increased R-Lath, and reduced plasma chol (16%) and LDL-chol (23%) concentrations.
2. SIMVA led to a slight, nonsignificant reduction in chol synthesis, a strong reduction in R_Lath and reduced plasma chol (28%) and LDL-chol (40%) concentrations.
3. EZE + SIMVA led to a strongly increased chol synthesis, a slightly reduced R-Lath and reduced plasma chol (40%) and LDL-chol (56%) concentrations.
4. Compared to omnivores, vegans had a much lower dietary chol intake, similar relative chol absorption rate, and lower plasma chol (18%) and LDL-chol (31%) concentrations but not a higher chol synthesis nor R_Lath.
5. The sum of daily chol synthesis and dietary chol absorption increased during EZE treatment and the EZE + SIMVA treatment. This was mentioned in the discussion of our previous paper without showing data [3]. The actual data are comprised here in Table 1.

This brings up the following questions:

1. When the sum of daily chol synthesis and dietary chol absorption increases, how can plasma chol concentrations be lowered?
2. How can an increased chol synthesis and a decreased R_Lath under EZE + SIMVA treatment be explained?
3. Why does chol synthesis increase in omnivore subjects under EZE treatment, but not during a vegan diet?

In this review we distinguish between chol synthesis as being the synthesis value calculated by the cholesterol balance procedure and* de novo* chol synthesis (DNCS). We will provide proof for the fact that chol synthesis is more than DNCS. In our view it includes a second flux of chol molecules extracted from blood by the liver, which we call the net plasma-liver chol exchange (PLCE) rate.

## 2. Model Establishment

**Figure 1:**
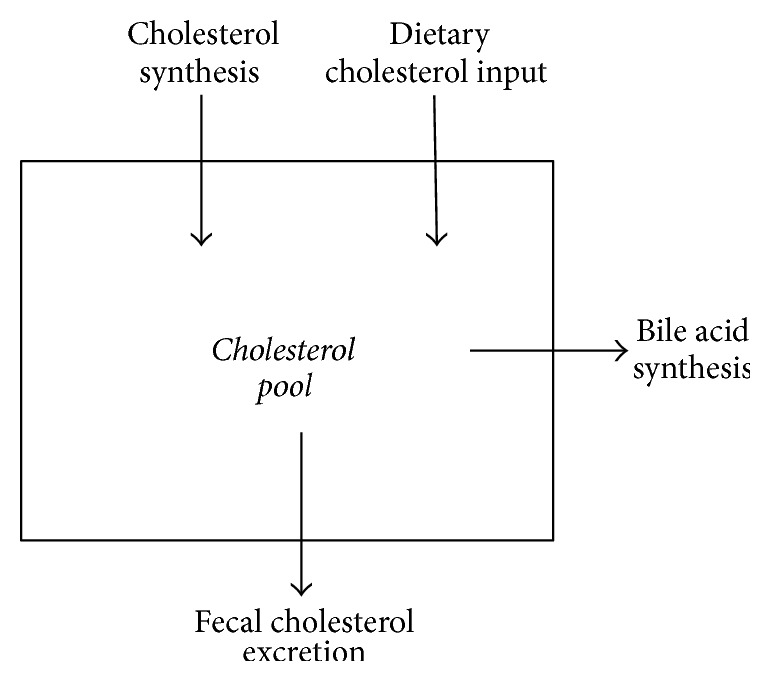
Principle of the cholesterol balance method.

To lower plasma chol concentrations, statins and EZE are thought to act synergistically by reducing the hepatic chol pool which leads to reduced VLDL secretion, increased LDL-apolipoprotein B-100 turnover, and upregulated LDL-receptor activity [5]. The general idea is that statins reduce and EZE increases DNCS, although the DNCS reducing effect of statins has recently been questioned [6]. As in our original studies [1, 2, 4], the daily chol synthesis rate (mg/d) is generally determined applying the cholesterol balance approach measuring the daily dietary chol input rate and the daily fecal excretion rate of chol metabolites and bile acids [7]. The chol synthesis rate is then calculated as the sum of daily excretion rate of neutral and acidic sterols minus the daily dietary intake of cholesterol (Figure 1). The daily excretion rate of acidic sterols represents bile acid synthesis. No alternative technique is available to measure the daily chol synthesis rate expressed as mg/d. Isotope techniques based on the measurement of the incorporation of an infused isotope labeled precursor (D_2_O, ^13^C-acetate) into plasma cholesterol present a relative value expressing the percentage of plasma chol that exists of newly synthesized chol produced within the infusion period. The principle of the classical cholesterol balance procedure is that an increased fecal loss of chol initiates an increased chol synthesis. In our alternative model we assume that not the increased fecal excretion but rather the diminished absorption rate is the trigger to increase both* de novo* synthesis and hepatic uptake of plasma cholesterol.

The fecal chol excretion consists of unabsorbed dietary (exogenous) cholesterol and endogenous chol: unabsorbed biliary chol, chol excreted via direct transintestinal excretion (TICE), and chol excreted via shedding of intestinal cells. In mice, it is estimated that TICE may represent about 30% of fecal total chol excretion. Intestinal cell shedding is estimated to represent about 20% of total fecal chol excretion. Related to endogenous chol these percentages will be higher, possibly together 70% of endogenous chol excretion. Thus a large part of chol excreted in feces is unrelated to dietary or biliary chol. Furthermore, malabsorbed dietary chol does not interfere with chol metabolism. Absorbed dietary chol molecules enter the pool of endogenous chol.

**Figure 2:**
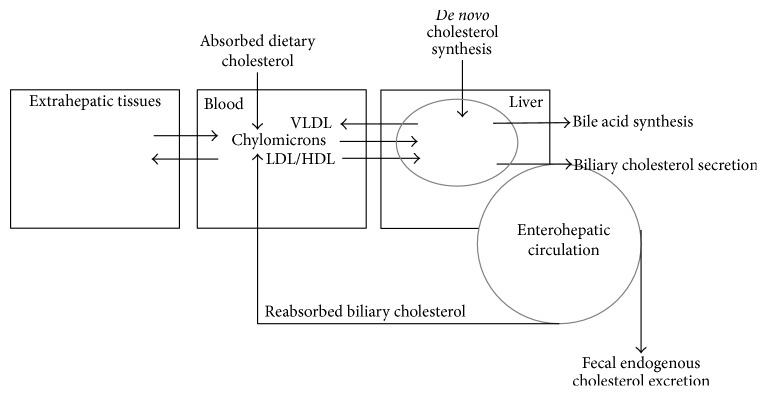
The central role of the liver in the regulation of endogenous cholesterol metabolism.

**Figure 3:**
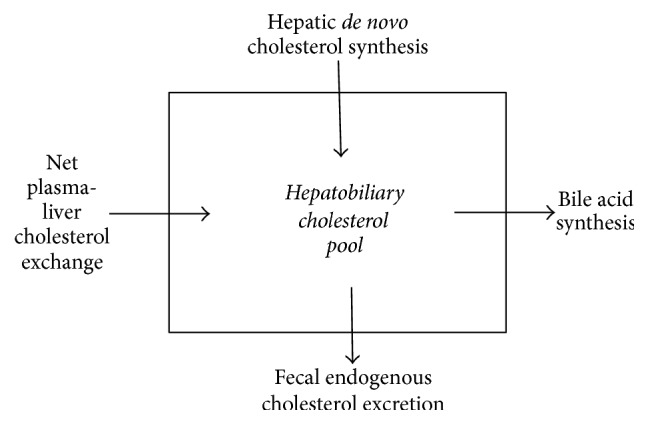
Alternative model of the cholesterol balance method.

According to compartmental analysis the endogenous chol pool contains three or even more pools with different turnover rates [8–10]. The pool with the highest turnover rate is called the rapidly exchangeable pool, which exchanges chol molecules with the slowly exchangeable pools. The pools cannot exactly be assigned to known organs. The definition of the rapidly exchangeable pool is subject of discussion. Generally, it is thought that this pool reflects the circulating hepatobiliary chol pool plus the blood chol pool, which exchanges with the slowly exchangeable pool present in the extrahepatic tissue membranes. It can also be argued that the fecal loss of endogenous chol molecules measured over a 72 h time period represents the turnover of chol present in the enterohepatic circulation. Here we propose a model, in which the liver acts as the central regulating organ and the hepatobiliary pool as the rapidly exchangeable pool, which exchanges molecules with the plasma lipoprotein system (Figure 2). The turnover is determined by the daily rate of biliary chol secreted into the intestine and its fractional rate of absorption as well as by bile acid synthesis. The fractional rate of chol absorption has been shown to be highly subject dependent and variable between about 20 and 80% applying the continuous feeding dual-isotope method [11, 12] and the blood based dual-isotope method [13]. Based on this model we hypothesize that chol synthesis determined with the cholesterol balance procedure contains two fluxes, that is, DNCS and the net exchange flux of chol molecules between plasma and liver (plasma-liver chol exchange flux, PLCE) as indicated in Figure 3. A reduction in fractional chol absorption in the intestine induced by EZE will increase the fecal exogenous and endogenous chol output via enhanced malabsorption of biliary and dietary chol and strongly reduce chylomicron chol uptake. The reduced chylomicron chol uptake, but not the enhanced fecal chol output contributes to regulation of hepatic chol fluxes. Bile acid synthesis remains unchanged [1–3]. As a result both hepatic chol synthesis and the net PLCE may increase. The latter factor explains the therapy induced reduction in plasma cholesterol. The order and degree with which both parameters increase are unknown.

## 3. Scientific Evidence for the Proposed Model

### 3.1. Whole Body Chol Synthesis versus Hepatic* De Novo* Synthesis

The net PLCE is the balance between chol molecules secreted by the liver into the blood (VLDL-chol secretion rate) and blood chol molecules extracted by the liver. The latter consist of the reverse chol transport taken up by the liver as LDL-chol or HDL-chol and of absorbed chol molecules arriving in chylomicrons secreted into blood by the intestine. The chylomicrons contain chol molecules from three different origins, that is, absorbed dietary chol, absorbed biliary chol, and possibly but unlikely absorbed excess chol synthesized in enterocytes. The direct transintestinal excretion of plasma derived chol molecules has been documented in mice [14–16] and must also be considered in the model. However, TICE represents plasma chol molecules that bypass the liver and are excreted directly into the feces. Therefore, no reabsorption of these molecules and no effects on the regulation of hepatic cholesterol synthesis must be considered.

Of importance in the discussion is the knowledge that cholesterol synthesis takes place in about all cells in the body, whereas regulation of plasma cholesterol levels occurs in the liver only. The basic knowledge about the distribution of chol synthesis over various tissues originates from the 1980s and 1990s and is limited to experimental animal models. In rodents [17, 18] the liver is the major site of chol synthesis (about 40–50%) followed by the skin (20%) and the intestine (10–20%). Unfortunately, human in vivo data are lacking. In the cynomolgus monkey Turley et al. [19] found that hepatic contribution is low, maybe 10–20%. In comparison intestinal chol synthesis appears two times stronger [19]. Otherwise, hepatic chol synthesis has been shown to be the target for regulation when the dietary chol intake is increased or absorption is impaired [20, 21]. Recent studies by Engelking et al. [22] established in mice that LDL-chol uptake and chol synthesis in the enterocyte are upregulated under EZE treatment. The question is what happens to the chol molecules derived from the upregulated synthesis and LDL uptake in the enterocyte. Are they incorporated into the membranes to compensate for the diminished intracellular pool size or are they partly incorporated in the chylomicrons? In the latter case it would imply that the daily absorbed amount of chol under EZE treatment is larger than expected from the dietary and biliary sources. We postulate that newly synthesized chol molecules in the enterocyte are not or only minimally absorbed. In case cholesterol synthesis in other extrahepatic tissues is increased in excess, this excess will be secreted into blood and be removed via hepatic extraction or TICE. In our model we define DNCS and position DNCS as hepatic* de novo* synthesis. This may be criticized. However, in the next chapter we will only discuss changes in chol synthesis induced by lipid lowering treatments. According to the literature [20, 21] we consider changes in DNCS to reflect changes in hepatic* de novo* synthesis. We also consider the change in R_Lath to reflect the change in hepatic DNCS. The arguments for this assumption are presented in Section 3.3.

### 3.2. Responses of Chol Metabolism to Plasma Chol Lowering Therapies

A major point of discussion concerns the different responses of cholesterol balance derived chol synthesis and R_Lath to EZE, SIMVA, and EZE + SIMVA treatments. EZE treatment leads to an enhanced fecal excretion of dietary chol, biliary chol, TICE [23], reverse transport chol [24–26] and as such an enhanced total fecal chol excretion. As reaction, increased chol synthesis has been described using the cholesterol balance procedure [1, 2], plasma lathosterol concentration (R_Lath) [3] and the deuterated water method [25]. The enhanced reverse chol transport via hepatic uptake of LDL-chol through an enhanced fractional turnover rate of LDL-apolipoprotein B-100 and increased LDL-receptor activity is associated with a decreased LDL-chol concentration but an unchanged turnover of extrahepatic tissue bound chol [25, 26]. Two explanations are possible. The first assumes that the reduced hepatic chol pool due to the strongly decreased chylomicron chol input is at first compensated by an insufficient increased net PLCE. As a second reaction hepatic DNCS increases to restore the hepatic chol pool. The second explanation might be that at first hepatic DNCS increases, however, insufficiently. Increased net PLCE could then complete the restoration of the hepatic chol pool. TICE under EZE treatment is not expected to be compensated by increased DNCS [27–29]. Intestinal cell shedding has to be compensated by cell renewal. The origin of membrane chol during cell renewal is unclear. Uptake from blood appears most likely since cells must be intact in order to initiate chol synthesis. Intestinal cell shedding is not expected to be increased during EZE treatment. EZE treatment results in a reduced biliary chol secretion [30, 31]. This means that the reduced reabsorption of biliary chol is not compensated by increased biliary secretion. Also SIMVA has been shown to reduce biliary chol secretion [32–34]. It may be concluded that biliary chol secretion is dependent on the hepatic chol pool size.

**Figure 4:**
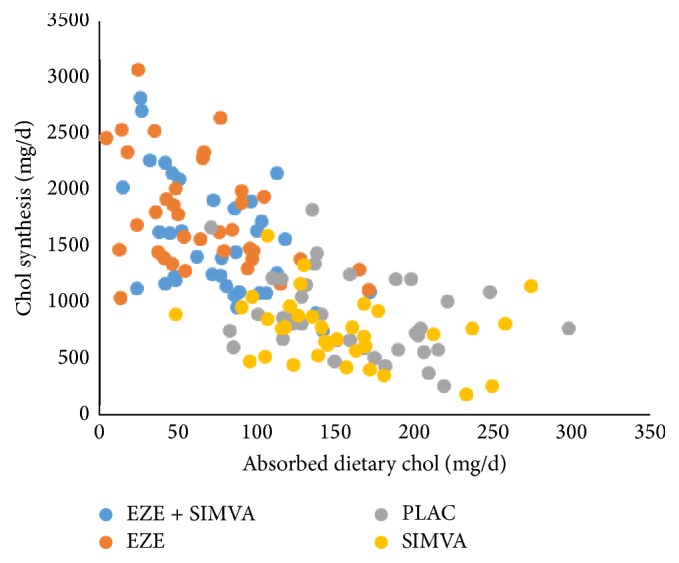
Inverse linear relationships between daily absorbed chol and chol synthesis as calculated by the cholesterol balance method. Data were obtained in the study published in [1].

From our original study [1], the following relationships could be obtained between daily absorbed dietary chol and chol synthesis calculated by the cholesterol balance method (Figure 4). These data confirm literature data [35, 36]. Applying our new model, this suggests that the sum of DNCS and net PLCE compensates for the reduction in chol absorption rate. Applying the two-tailed Spearman correlation matrix, significant negative relations were found in omnivores under PLAC treatment (*r* = −0.3695, *p* = 0.0244), EZE treatment (*r* = −0.3894, *p* = 0.0189), and EZE + SIMVA treatment (*r* = −0.4858, *p* = 0.0027). Under SIMVA treatment the relation was nearly significant (*r* = −0.3142, *p* = 0.0661). For the vegans the correlation was not significant. For omnivore subjects under all treatment conditions no significant correlations were found between R_Lath as marker for DNCS and R_Camp as marker for cholesterol absorption. This may imply that DNCS is not a regulated parameter but is upregulated only when net PLCE cannot be increased sufficiently.

**Figure 5:**
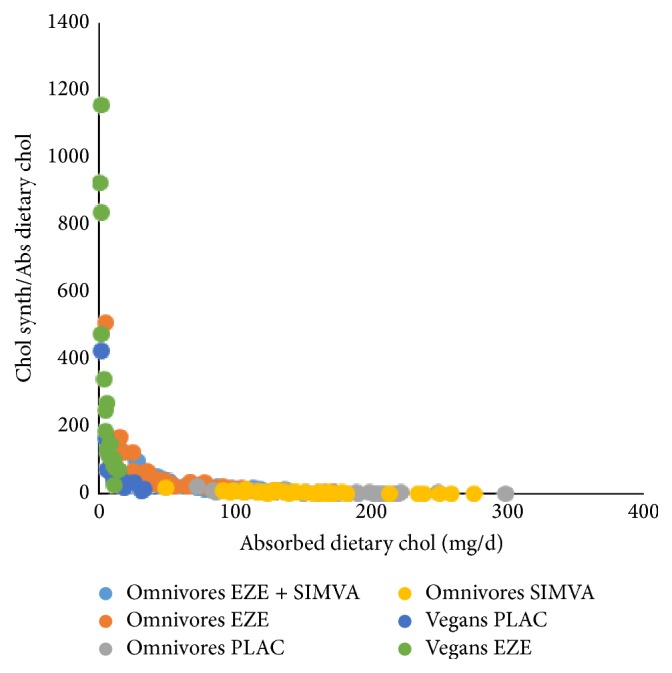
The sensitivity of chol synthesis, calculated by the cholesterol balance method, to react on the level of dietary chol absorption. Data were obtained in the studies published as in [1] and [4].

**Figure 6:**
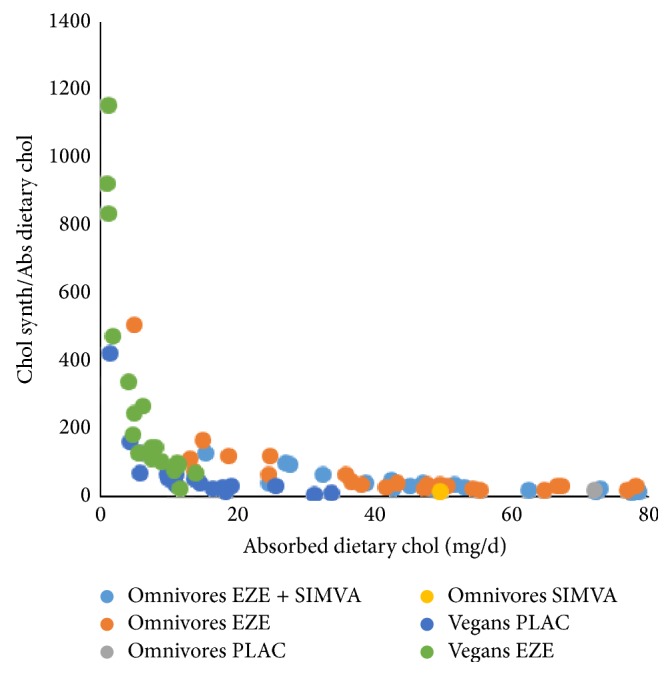
The sensitivity of chol synthesis, calculated by the cholesterol balance method, to react on the level of dietary chol absorption. Data were obtained in the studies published as in [1] and [4].

Interestingly, when the chol synthesis/absorbed dietary chol ratio is plotted against the absorbed dietary chol, extremely significant negative exponential correlations were found (Figures 5 and 6). Two-tailed Spearman correlation analysis resulted in the following correlation parameters: PLAC *r* = −0.777, *p* < 0.0001; EZE *r* = −0.9524, *p* < 0.0001; SIMVA *r* = −0.7925, *p* < 0.0001; EZE + SIMVA *r* = −0.9108, *p* < 0.0001; vegans *r* = −0.9375, *p* < 0.0001; vegans under EZE treatment *r* = −0.9546, *p* < 0.0001. Apparently chol synthesis becomes accelerated when the flux of absorbed dietary chol is below 50 mg/d. The strongest increases in chol synthesis associated with decreasing dietary chol absorption are observed under EZE treatment. The increase under dietary intake restriction in vegans is only mild. Unfortunately, it cannot be clarified whether the strongest increases of chol synthesis are caused by increased DNCS, by increased net PLCE, or by both. Most likely, in subjects with a low rate of absorbed dietary chol due to a low dietary chol intake or a naturally low fractional absorption rate, net PLCE is increased first. Under EZE treatment but not during a vegan diet DNCS is increased as indicated by R_Lath. Thus the increased DNCS under EZE treatment may possibly be explained as a drug induced hepatic effect and not as a consequence of the low dietary chol absorption rate. This topic will be discussed in more detail in Section 3.4. when chol fluxes in vegans are compared with those in omnivores under PLAC and EZE treatment. During the combination treatment (EZE + SIMVA) in our original study [1] the doses of EZE and SIMVA were 10 mg and 20 mg respectively. The low dose of SIMVA enabled to compensate the increase in EZE induced DNCS as indicated by the R_Lath value (56%) and to increase the plasma LDL-chol lowering effect from 20 to 55%. The plasma chol lowering effect during the combination therapy, when DNCS is unchanged, must be initiated by the increased PLCE only.

As mentioned before no alternative method is available to determine chol synthesis expressed as mg/d. No methods are available to determine DNCS or net PLCE. Therefore DNCS and net PLCE cannot be differentiated. A relative change in DNCS can be predicted by the relative change in R_Lath. But in order to translate this change into an absolute change in DNCS, a distribution between DNCS and net PLCE in the untreated state must be made. The change in the calculated DNCS then remains dependent on the original distribution assumption. However, the change in DNCS relative to the untreated value becomes independent of the distribution assumption and is solely dependent on R_Lath.

### 3.3. R_Lath as a Marker for Hepatic DNCS

In this discussion we hypothesize that R_Lath is a marker for DNCS. This is mainly based on the observations where R_Lath reflects expected changes in DNCS much better than the cholesterol balance derived synthesis value [3] under EZE, SIMVA, and in particular EZE + SIMVA treatment. It is also considered to be a selective marker for hepatic DNCS. This can be defended by the following arguments. It appears logical to assume that chol synthesis in a tissue is increased only when the intracellular pool is diminished and alternative chol sources are not available to restore the situation. The newly synthesized chol will be incorporated in the cell membranes and stay inside the cell. Only when excess chol exists in the cell, chol molecules will be transported out of the cell into the blood stream. This is not the case during EZE and SIMVA treatments. The liver is a unique tissue since it is able to freely transfer chol into blood and bile and to synthesize chol and bile acids. That enables the liver to regulate plasma chol levels. It may be expected that also hepatic cholesterol precursors such as lathosterol and desmosterol are freely exchangeable between the liver cell and plasma. Lathosterol and desmosterol produced in other tissues will be further metabolized to chol without exchange with blood. Therefore, plasma cholesterol precursors as markers of cholesterol synthesis may be considered hepatic specific. This is supported by early observations that plasma lathosterol is highly correlated with HMG-CoA Reductase activity in the liver [37] and that hepatic synthesis is the target of regulation when chol intake is increased or chol absorption is impaired [20, 21]. Additional support is provided by studies showing reduced plasma cholesterol precursor concentrations in liver disease and an increase of these marker values after liver transplantation [38–40]. Also in vitro studies showed a reduction in lathosterol synthesis in liver cells in which chol synthesis was inhibited using statins or cholesterol containing plasma [41]. Furthermore, it was shown that the reduction of plasma lathosterol by pravastatin was lower in carriers of the SLCO1B1 haplotype *∗*17 in which hepatic uptake of statins is reduced [42]. An interesting question in this respect is whether organs differentiate between the Bloch and Kandutsch-Russell pathways converting lanosterol to cholesterol. Such a differentiation would affect the diagnostic value of desmosterol (Bloch pathway) and lathosterol (Kandutsch-Russell pathway) as markers for chol synthesis. Mitsche et al. [43] studied this in cultured cells of mouse tissues and differentiated between high (>60%, liver, kidney, adipose, spleen, testes, adrenal) and low (<40%, muscle, heart, brain, preputial, skin) Bloch metabolizing tissues. Unfortunately, the intestine was not studied. Hepatic chol synthesis used a mixture of the Bloch pathway (60%) and Kandutsch-Russell pathway (40%). In no organ a selective use of either the Bloch pathway or the Kandutsch-Russell pathway was found.

### 3.4. Comparison of Chol Fluxes in Vegans and Omnivores during EZE Treatment

An interesting point of discussion is the observation [3, 4] that chol synthesis in vegans determined with the cholesterol balance method as well as via R_Lath is not higher than in omnivores. Vegans have an extreme low dietary chol intake, but a normal fractional absorption rate [4]. Both fecal chol excretion rate and dietary chol absorption rate are low. Apparently, this does not lead to an increased chol synthesis rate. The plasma chol concentrations in vegans are lower than in omnivores and comparable to omnivore subjects on EZE treatment [1, 4]. This suggests that the plasma chol lowering effect in vegans is solely due to an increased net PLCE. Vegans and omnivores under EZE treatment differ largely in the fecal chol excretion rate but share low daily dietary chol absorption rates. The daily amount of absorbed dietary chol was even lower in the vegans [3] (average 14 mg/d) than in the omnivores under EZE treatment [1] (average 66 mg/d). A lower R_Lath in vegans (average 1.1) compared to omnivores on EZE (average 2.5) [3] suggests that* de novo* hepatic chol synthesis is lower in the vegans. The difference between the chol synthesis rates calculated by the cholesterol balance method for the omnivores on EZE (average 1851 mg/d) [1] and for vegans (average 595 mg/d) [4] confirms the difference between the R_Lath values. The combination of data suggests that both DNCS and net PLCE are much lower in vegans compared with omnivores on EZE resulting in similar low plasma chol and LDL-chol concentrations. One piece of information is missing in this comparison, that is, the daily flux of absorbed biliary chol. When biliary chol secretion is discussed, hepatic chol secretion into bile is meant, which is normally measured in mice applying cannulation of the common bile duct and collecting hepatic bile during a certain time period. In humans, a change in biliary chol secretion is concluded when the chol concentration and in particular the chol/bile acids ratio changed in duodenally aspirated bile. For the flux of absorbed biliary chol the frequency and extent of gallbladder contraction are important parameters. This flux is low in omnivores under EZE treatment, due to the low fractional absorption rate. Interestingly in mice EZE has been shown to improve gallbladder motility [44], possibly as an attempt to compensate the reduced intestinal chol flux. This finding, in combination with a lower biliary chol secretion explains the observed reduction in gallstone incidence during EZE treatment [30, 31]. In vegans the fractional absorption rate is normal and the daily flux of absorbed biliary chol may be normal when biliary chol secretion and the frequency and extent of gallbladder contraction are normal. Unfortunately detailed information on biliary chol secretion and gallbladder dynamics in vegans is missing in the literature. Most likely the sum of daily absorbed dietary plus biliary chol is larger in vegans than in omnivores on EZE treatment. Since the daily flux of biliary chol through the intestine is much larger than the flux of dietary chol, this might explain why chol synthesis is not increased in vegans. More detailed knowledge on biliary chol secretion and gallbladder motility in humans on EZE treatment and on a vegan diet would be very helpful in clarifying the role of biliary chol absorption in the regulation of DNCS.

It may also be speculated that the increases of chol synthesis and R_Lath under EZE treatment are induced by EZE as a direct hepatic drug effect. Pharmacological studies have shown that EZE is readily absorbed in the small intestine and undergoes enterohepatic circulation [45]. Furthermore, EZE is partly converted to its glucuronide conjugate [45–47]. EZE is excreted via feces and urine. The enterohepatic cycling implies that a continuous flux of EZE molecules is being transported through the liver. The Niemann-Pick C1-Like 1 (NPC1L1) transporter in the enterocyte is the target of EZE action. However, NPC1L1 is also present on the canalicular membrane in the liver [48]. Its function there is thought to be to transport biliary secreted chol back to the liver [49, 50]. Biliary chol secretion is balanced by the actions of NPC1L1 and ABCG5/ABCG8 that activates biliary secretion. It may be expected that EZE suppresses NPC1L1 at the canalicular membrane, which should lead to an increased biliary chol secretion. However, on the contrary, biliary chol secretion has been shown to be reduced under EZE treatment [30, 31], which makes EZE a very interesting drug to treat gallstone formation. Therefore, it must be assumed that EZE may exert a so far unknown hepatic interaction leading to increased DNCS and reduced biliary chol secretion. This may or may not be related to the observation of Yamamura et al. [51] that EZE treatment is associated with increased autophagy in human hepatocytes.

The paper of Clarenbach et al. [4] shows that EZE treatment of vegans leads to a further reduction of the low daily absorbed dietary chol from 14 to 6 mg/d. In Figures 5 and 6 it can be seen that the exponential increase in chol synthesis in untreated vegans is mild but that EZE treatment leads to a tremendous increase in chol synthesis. In mean values chol synthesis increased 72% from 595 to 1022 mg/d, whereas R_Lath increased 55%. In omnivores Sudhop et al. [1] found a 110% increase in chol synthesis and a 56% increase in R_Lath [3] during EZE treatment. The data in both populations suggest that EZE leads to an increase in both DNCS and net PLCE. The data in EZE treated vegans supports the hypothesis that the increased DNCS may be a specific EZE effect and not an effect of low chol absorption.

## 4. Summary and Conclusions

We presented an alternative view on the interpretation of cholesterol balance derived chol synthesis data and those of surrogate markers of chol synthesis, particularly in view of plasma cholesterol lowering therapies. In the traditional whole body model for the cholesterol balance procedure an increased fecal excretion of endogenous chol is translated into an increased whole body chol synthesis. However, fecal cholesterol has various origins and not all are affected by lipid lowering therapy. Thus cholesterol balance derived chol synthesis data must be interpreted with caution during lipid lowering therapies. Regulation of DNCS takes place in the liver. The chylomicron transported chol absorbed from the intestine contributes to hepatic chol regulation of DNCS, not the chol excreted in the feces. Therefore, the original whole body cholesterol balance model was replaced by a model in which the enterohepatic chol pool is defined as the rapidly exchangeable chol pool and the liver as the central regulating system where all endogenous chol influxes and effluxes come together. The turnover of this pool is determined by the biliary chol secretion rate, gallbladder motility, the fractional chol absorption rate and by bile acid synthesis. In kinetic terms the hepatic chol influx can be described by the sum of hepatic* de novo* synthesis (DNCS) and net plasma-liver cholesterol exchange rate (PLCE). In case of ezetimibe treatment, the total chol absorption rate and thus the net daily amount of absorbed chol introduced to the liver via chylomicrons is strongly reduced and causes the hepatic chol pool to diminish strongly. This leads to enhancement of the net PLCE and DNCS in an unknown order and degree. Data suggest that enhanced net PLCE is the primary response and enhanced DNCS the second response. Statins lead to a decreased hepatic DNCS, a diminished hepatic chol pool and increased net PLCE. Combined statin/ezetimibe treatment leads to a net unchanged DNCS, diminished hepatic chol pool and increased net PLCE. Changes in DNCS during lipid lowering treatments are changes in hepatic DNCS, that are reflected by changes of plasma R_Lath. Therefore, R_Lath may be considered as a useful marker for hepatic DNCS. Increased DNCS during ezetimibe treatment in omnivores and vegans, but no increased DNCS during a vegan diet alone suggests a crucial role of the flux of absorbed biliary chol which is most likely high in untreated vegans and low in ezetimibe treated subjects. A direct hepatic drug effect induced by ezetimibe cannot be excluded, while ezetimibe is effectively absorbed and undergoes enterohepatic cycling.

## Figures and Tables

**Table 1:** Total daily cholesterol input calculated as the sum of daily absorbed cholesterol and cholesterol synthesis measured by the cholesterol balance method. Original data have been published [1].

Treatment	Chol synthesis	Absorbed dietary chol	Total chol input
	mg/day	mg/day	mg/day
PLAC	868 ± 358	162 ± 51	1004 ± 312
EZE	1846 ± 737^*∗∗∗*^	66 ± 41^*∗∗∗*^	1821 ± 474^*∗∗∗*^
SIMVA	747 ± 295	159 ± 59	899 ± 290^*∗*^
EZE + SIMVA	1607 ± 648^*∗∗∗*^	76 ± 37^*∗∗∗*^	1616 ± 493^*∗∗∗*^

^*∗∗∗*^ *p* < 0.0001, ^*∗*^*p* < 0.01.

## Abbreviations

ABCG5/ABCG8: Adenosine triphosphate binding cassette (ABC) heterodimer transporters
APO B100: Apolipoprotein B100
Chol: Cholesterol
DNCS: * De novo* cholesterol synthesis
EZE: Ezetimibe
HMG-CoA: 3-Hydroxy-3-methyl-glutaryl-coenzyme A
LDL: Low density lipoprotein
NPC1L1: Niemann-Pick C1-like 1
PLCE: Plasma-liver cholesterol exchange
R_Lath: Plasma lathosterol/cholesterol ratio
SIMVA: Simvastatin
TICE: Direct transintestinal cholesterol excretion.
